# Psychological stress and risk of incident atrial fibrillation in men and women with known atrial fibrillation genetic risk scores

**DOI:** 10.1038/srep42613

**Published:** 2017-02-14

**Authors:** Thomas Svensson, Mariusz Kitlinski, Gunnar Engström, Olle Melander

**Affiliations:** 1 Department of Clinical Sciences, Lund University, Skåne University Hospital, SE 205 02 Malmö, Sweden; 2 Department of Global Health Policy, Graduate School of Medicine, The University of Tokyo, 7-3-1 Hongo, Bunkyo-ku, Tokyo 113-0033 Japan; 3 Department of Neuropsychiatry, Keio University School of Medicine, 35 Shinanomachi, Shinjuku-ku, Tokyo 160-8582, Japan; 4 Department of Cardiology, Skåne University Hospital, Malmö, Sweden; 5 Department of Internal Medicine, Skåne University Hospital, Malmö, Sweden

## Abstract

Psychological stress has been reported as a possible trigger of atrial fibrillation (AF). No studies have investigated whether any association between stress and AF could be modified by genetic susceptibility to AF (AF-genetic risk score (AF-GRS)). 8765 men and 13,543 women from the Malmö Diet Cancer Study, a population-based cohort, were included in the analyses. A variable representing stress was constructed from questions measuring job strain, and from one question assessing non-occupational stress. Cox proportional hazards regression models were adjusted for known covariates of AF. Mean follow-up times and number of recorded incident AF were 14.2 years and 1116 events for men, and 15.1 years and 932 events for women. Among women, high stress was associated with AF in the age adjusted model (hazard ratio [HR], 1.22; 95% confidence interval [CI], 1.01–1.47) but not following multivariable adjustment (HR, 1.15; 95% CI, 0.95–1.39). Stress was not associated with incident AF in men. AF-GRS was significantly associated with incident AF for both genders. Stress did not interact significantly with genetic susceptibility to AF in men or women. Chronic stress is not associated with long-term incident hospital diagnosed AF. This association does not appear to be modified by genetic susceptibility to AF.

Psychological stress has been discussed and reported as a possible trigger of atrial fibrillation (AF) for a number of decades^1^,^2^,^3^. Psychological variables independently predict early development of AF^4^,^5^, and job strain is marginally associated with an increased risk of AF in men^6^, yet there is no epidemiologic evidence of any direct association between perceived psychological stress and long-term incident AF in large general populations.

The explanatory pathways through which psychological stress may affect the heart and possibly elicit AF involve the autonomic nervous system^7^ and the hypothalamus-pituitary-adrenal (HPA) axis^8^. The neurogenic effect of stress produces electrophysiological changes in the heart, in particular on the function of the sinus node and AV node^9^. Genetic factors have been suggested to determine sympathovagal cardiac control^10^ and individual cardiovascular stress reactivity^11^, thereby indicating that susceptibility to stress-induced AF may differ between individuals. Recently an AF genetic risk score (AF-GRS) was found to predict incident AF in a large general population cohort^12^. Given the importance of stress exposure in combination with the possibility of genetically determined individual cardiovascular stress reactivity^11^, a genetic risk score could possibly modify the impact of psychological stress on the development of AF. The purpose of the current study was thus to investigate if there is any association between psychological stress and long-term incident AF in a population cohort, and for the first time to elucidate whether any association between psychological stress and long-term incident AF varies by genetic susceptibility to AF. We hypothesized that psychological stress would further increase the risk of an event in those with the highest genetic risk of AF.

## Methods

The Malmö Diet and Cancer (MDC) study is a population based prospective study. Details of the study have been described elsewhere^13^. Briefly, men and women aged 45–73 years were selected at random from a population of approximately 230,000 in the city of Malmö, Sweden and recruited for a baseline examination between the years 1991–1996. In addition to anthropometric data and blood samples, participants were asked to fill in a detailed questionnaire on heredity, socioeconomic variables, social network, occupation, physical activity, alcohol consumption, smoking, diseases and medication.

The study population identified 30,447 individuals at baseline. We excluded participants with a history of AF (n = 312) and those for whom we had incomplete information on stress (n = 5557), insufficient information of genotyping (n = 2269) and who were lost to follow-up (n = 1). A total of 8765 men and 13,543 women were included in the analyses.

The MDC study was approved by the ethics committee at Lund University. All participants provided written informed consent and all methods were performed in accordance with the relevant guidelines and regulations.

### Psychological stress

A variable representing psychological stress was constructed from questions on psychosocial work characteristics measuring job strain, and from one question assessing non-occupational stress.

Job strain was assessed using items in the validated Swedish version^14^ of Karasek’s^15^ and Theorell’s^16^ Demand-Control Model. The Psychological Demands (PD) and the Decision Latitude (DL) subscales in the questionnaire consisted of five and six items respectively. Each item was assessed using a 4-point Likert scale, allowing summation of the scores for each subscale. Scores ranged from 5–20 for PD, and 6–24 for DL. In accordance with how the scale has been used previously^16^, none of the answers were weighted. Job strain was calculated by dividing PD with DL, and the result was dichotomised at the median to allow for a ‘low strain’ and a ‘high strain’ group. The job strain questionnaire has previously been used in cardiovascular research^17^.

Non-occupational stress was assessed using the following yes/no question: ‘*Have you lately suffered from stress or mental pressure because of problems or demands not related to your work?*’ An answer of ‘yes’ indicated high stress whereas the opposite was true for those who answered ‘no’. Single questions to assess the association between perceived mental stress and cardiovascular end points have been used previously in large prospective studies^18^.

By combining the dichotomised variables job strain and non-occupational stress, a categorical variable was created representing low, intermediate, and high psychological stress. Individuals with low strain and low stress were considered to have low psychological stress; those with low strain and high stress or high strain and low stress were considered to have intermediate psychological stress, and individuals with high strain and high stress were assigned to a high psychological stress group. Cronbach’s alpha for the 12 items assessing stress in the present study was 0.69.

### Genetic risk score

An Atrial Fibrillation Genetic Risk Score (AF-GRS) comprised of 12 SNPs has been shown to predict AF^12^. Each of the 12 SNPs is associated with AF at genome-wide significance levels^19^,^20^,^21^,^22^,^23^,^24^. In contrast to the study by Tada *et al*.^12^, the risk score in the present study focused on risk alleles instead of minor alleles and thus carried a minimum value of 0. The AF-GRS was calculated by multiplying the number of carried risk alleles (0/1/2) with the natural logarithm transformed risk estimate for that allele. The products were subsequently summed and divided by the total number of genotyped alleles for each individual.

### Genotyping

A multiplex method combining polymerase chain reaction, allele-specific oligonucleotide ligation assays, and hybridization to oligonucleotides coupled to Luminex 100TM xMAP microspheres (Luminex, Austin, TX) was used for the genotyping of MDC study participants^25^.

### End points

The primary end point of this study was incident AF defined according to diagnosis codes of three revisions of the International Classification of Diseases (ICD), 427.92 (ICD-8), 427D (ICD-9), and I48 (ICD-10). All prevalent cases and incident AF events were identified through linkage of a 10-digit national personal identification number with two registries validated for classification of outcomes and administered by the Swedish National Board of Health and Welfare: the Swedish National Discharge Registry, and the Swedish National Cause of Death Registry. The validity of AF diagnosis in these registers is high^26^. Participants were followed from starting point until December 31, 2010, with person-years calculated from starting point to the date incident AF, loss to follow-up, or end of follow-up period, whichever came first.

### Statistical analyses

To determine the possibility of individual SNPs clustering within stress levels, the cross-sectional association between stress level and minor allele carrier status of each of the 12 SNPs included in the AF-GRS was investigated using univariable logistic regression analyses.

The association between AF-GRS and stress, and incident AF was estimated using Cox proportional hazards regression. The gender and age adjusted models were adjusted for gender and age (continuous) at starting point. Multivariable models were further adjusted for education (elementary school or higher than elementary school), socioeconomic index (SEI), smoking (never-, past- and current smoker), alcohol consumption (none, 1–15.2, and ≥15.2 g ethanol/day), prevalent diabetes mellitus, coronary events, heart failure, Body Mass Index (BMI) in kg/m^2^ (continuous), and hypertension.

Effect modification was determined by including an interaction term between stress level and 1) AF-GRS, and 2) SNPs significantly associated with stress in the cross-sectional analyses, respectively. The multivariable models including the interaction term were subsequently compared to those without this term by using the Likelihood ratio test.

SEI was categorized according to the Swedish socioeconomic classification^27^ (manual worker, low and intermediate level non-manual worker, higher level non-manual worker, other (self-employed incl. farmers), and unemployed). Prevalent diabetes mellitus was defined as self-reported physician diagnosed diabetes, use of antidiabetic medication, fasting blood glucose ≥6.1 mmol/l, or belonging to local or national diabetes registries. Hypertension was defined as systolic blood pressure ≥140 mmHg, diastolic blood pressure ≥90 mmHg or use of antihypertensive medication. Missing data were addressed through the construction of dummy variables.

Sensitivity analyses excluded individuals using beta receptor antagonists at the start of the study, given that the sympathetic nervous system is a candidate pathway for the effects of stress on the heart^8^. Additional sensitivity analyses were conducted due to the use of a composite stress variable; 1) excluding individuals who were unemployed at the time of filling in the questionnaire, 2) using only job strain as the main exposure, and 3) using only non-occupational stress as the main exposure. Analyses were stratified according to gender owing to reported gender differences in stress response^28^.

All statistical analyses were performed using SAS (SAS software version 9.3; SAS institute, Inc., Gary, NC). The significance level was set as P < 0.05.

### Data Availability

Due to ethical and legal restrictions related to the Swedish Biobanks in Medical Care Act (2002:297) and the Personal Data Act (1998:204), data are available upon request from the data access group of Malmö Diet and Cancer study by contacting Anders Dahlin (anders.dahlin@med.lu.se).

## Results

A comparison of the baseline characteristics of men (n = 2004) and women (n = 3011) who did not respond to questions on stress but had complete information on genotyping with the men and women included in our study showed that non-responders to stress were less likely to be unemployed (both men and women). Male non-responders were younger and more likely to be lower and intermediate non-manual workers, and never drinkers whereas female non-responders were more likely to be manual workers, have lower education, be never smokers, and less likely to be heavy drinkers (data not shown).

Three SNPs were associated with stress level in the univariate analyses (Supplementary Table S1). Rs3903239 (PRRX1) was inversely associated with intermediate stress level in women (odds ratio [OR], 0.91; 95% confidence interval [CI], 0.84–0.99), rs3807989 (CAV1) was positively associated with intermediate stress in men (OR, 1.13; 95% confidence interval [CI], 1.00–1.28), and rs1152591 (SYNE2) was inversely associated with high stress in combined analyses (OR, 0.88; 95% confidence interval [CI], 0.80–0.96), and with intermediate (OR, 0.91; 95% confidence interval [CI], 0.84–1.00) and high stress (OR, 0.86; 95% confidence interval [CI], 0.77–0.96) in women, respectively. The interactions between psychological stress and risk allele carrier status for each of the three SNPs associated with stress were non-significant in combined analyses on incident AF (rs1152591 ([SYNE2]), and in stratified analyses for men (rs3807989 [CAV1]) and women, (rs3903239 [PRRX1], rs1152591 [SYNE2]), respectively (data not shown).

The 8765 men and 13,543 women participating in this study were followed for an average of 14.2 and 15.1 years respectively. During follow-up there were 1116 recorded AF events in men and 932 recorded events in women. Baseline characteristics within each stress category according to gender are provided in Table 1. Both men and women who reported low psychological stress were older, more likely to belong to a higher SEI category, more likely to be never/past smokers, and more likely to consume alcohol. Men and women who belonged to the high stress group were more likely to be unemployed, current smokers, and non-responders to questions on alcohol consumption.

In the age and gender-adjusted model for analyses on men and women, high psychological stress was associated with AF (hazard ratio [HR], 1.16; 95% confidence interval [CI], 1.01–1.32) although this result was attenuated following multivariable adjustment (Table 2).

In gender stratified analyses, high psychological stress was associated with incident AF in women only in the age adjusted models (HR, 1.22; 95% CI, 1.01–1.47) (Table 2). This association was no longer statistically significant following multivariable adjustment (HR, 1.15; 95% CI, 0.95–1.39).

When compared with the lowest AF-GRS quartile in our age and gender adjusted models, quartiles 3–4 were associated with an increased risk of incident AF. This association did not change in the multivariable adjusted models with significantly increased risks of AF for individuals in the third (HR, 1.36; 95% CI, 1.19–1.55) and fourth (HR, 1.72; 95% CI, 1.52–1.95) quartiles.

In gender stratified analyses and when compared with the lowest AF-GRS quartile in our respective age adjusted models, quartiles 2–4 were associated with an increased risk of incident AF in men whereas quartiles 3–4 were associated with an increased risk of AF in women respectively. Multivariable adjustment did not change the association between AF-GRS and the end point; men in AF-GRS quartile 2 (HR, 1.25; 95% CI, 1.05–1.50), 3 (HR, 1.40; 95% CI, 1.17–1.67), and 4 (HR, 1.75; 95% CI, 1.47–2.07), and women in quartile 3 (HR, 1.31; 95% CI, 1.09–1.59) and 4 (HR, 1.68; 95% CI, 1.40–2.01) remained at a significantly increased risk of incident AF.

The interactions between psychological stress and AF-GRS in the combined analyses as well as in the gender stratified analyses were non-significant (Table 3).

### Sensitivity analyses

Sensitivity analyses which excluded individuals using beta receptor antagonists at the start of the study (men: n = 1031; women: n = 1191) did not markedly change the results of the main associations or of stress – AF-GRS interactions (Supplementary Tables S2 and S3).

Sensitivity analyses which utilized only job strain and non-occupational stress, respectively, as the main exposures did not change the overall interaction results (Supplementary Tables S4–S7). Additional sensitivity analyses which excluded unemployed individuals did not change the overall results (data not shown).

## Discussion

This study is to the best of our knowledge the first of its kind to investigate the potentially modifying effect of chronic psychological stress on a genetic risk score known to predict incident AF in men and women. Although a number of case reports have highlighted that stress has acted as a trigger of AF episodes on an individual level^1^,^2^,^3^, the results of this study show an association between stress and AF in the age and gender adjusted models for the whole population, and only for women in gender stratified analyses. Controlling for additional modifiers attenuates any significant association between chronic stress and long-term incident hospital diagnosed AF on a population level. Results indicate that although AF-GRS is strongly associated with AF in both genders, the association between psychological stress and AF does not appear to be modified by genetic susceptibility to AF. However, despite the lack of significant overall gene-environment interactions, findings are suggestive of gender specific stress responsivity according to AF-GRS quartiles in the multivariable adjusted models. Compared to men with low genetic risk and low stress, the direction of results for men with low genetic risk and intermediate and high stress, respectively, point toward a reduced risk of AF.

Psychological stress was assessed in this study using established questions based on the job strain model, and a question about non-occupational stress, and the resulting variable divided into three stress categories. As expected, men and women with low stress were characterized by older age and higher SEI, whereas those with high stress were characterized by unemployment and current smoking status. In addition, men with low stress had higher education than the other stress categories, and women with low stress had the lowest BMI. These findings are in accordance with previous studies which have linked high stress with younger age^29^, lower SEI^29^, unemployment^29^, lower education^29^, impaired smoking cessation^30^ and increased BMI in women^30^.

In order to discuss our results in the context of other findings on the association between psychological predictors and AF, key differences between the two main autonomic pathways that drive episodes of neurogenic AF need to be highlighted. Vagally mediated AF is elicited by relaxation which follows periods of stress and is seen in young individuals, predominantly males, without any structural heart disease whereas adrenergically induced AF occurs during periods of sympathetic activity, e.g. psychological stress, and is less frequent than its vagal counterpart but more often seen in patients with heart disease^31^. These distinctions between the two major pathways of neurogenic AF may partly help to explain the difference between our own results and the findings by Eaker *et al*.^4^,^5^ who identified anger, hostility and tension as independent predictors of AF in men. First, the main exposures of job strain and non-occupational stress in our study represent different psychological entities compared to anger, hostility, and tension. Moreover, whereas the mean age of our study population was 59.6 and 57.2 for men and women respectively, the Framingham Offspring Study had much younger participants, some as young as 18, and an overall population mean age of 48.5. Such population characteristics would have increased the possibility to detect the more prevalent vagally mediated neurogenic AF which, in accordance with their study findings, also happens to be more common among men. The higher mean age of our study population and the stress variable used here would instead be more appropriate to predict adrenergically induced AF. Unfortunately, this type of neurogenic AF is rare and occurs predominantly in those with structural heart disease which could explain the lack of any associations in our study.

The AF-GRS in this study was comprised of 12 individual genetic variants and is a strong predictor of incident AF^12^. The basis for testing the interaction between AF-GRS and psychological stress is that that susceptibility to stress-induced AF may differ between individuals, and that genetic factors have been proposed to determine individual cardiovascular stress reactivity^11^. Moreover, individuals with panic disorder may have deregulated autonomic arousal and increased vagal withdrawal^32^. It is possible that similar situations could occur in predisposed individuals during prolonged periods of chronic stress. Such inter-individual differences in stress reactivity warrant investigation. Although the results of our study indicate that a 12 SNP AF-GRS cannot be directly linked with stress-induced AF on a population level, we cannot, however, entirely exclude the possibility that the AF-GRS could serve as an indicator of future structural heart changes.

Our sensitivity analyses excluded individuals who used beta receptor antagonists. Although interactions were non-significant, results from these analyses indicate that for women in the top AF-GRS quartile, the risk of event increased from 1.85-fold at low and intermediate stress to almost 2.4-fold at high stress – a risk ratio of approximately 1.3. The corresponding risk ratio for the group which included users of beta receptor antagonists was 1.1. These results would thus be in line with findings suggesting an adrenergically induced neurogenic AF in which treatment with beta receptor antagonists is indicated. Despite the lack of any significant overall interaction in our study, the exclusion of subjects in this sensitivity analyses resulted in comparatively fewer cases in the high stress group with a subsequent loss of statistical power. Further studies on the association between psychological stress and AF are warranted and future studies should take into account both population age and gender differences in addition to consider the possibility of a protective effect by beta antagonists on the arrhythmogenic effects of psychological stress in older women.

There are limitations to the study that need to be acknowledged: First, psychological stress was measured at only one point. Stress is an exposure that may change over time and should have repeated measurements in a longitudinal study with extended follow-up times. There is thus a possibility of random misclassification and an underestimation of exposure. Moreover, a variable such as psychological stress is likely to be associated with residual confounding. There are also differences in certain baseline characteristics when comparing participants and non-responders to questions on stress. However, when including non-responders to questions on stress in the final models of our main gender stratified analyses, additional analyses did not reveal any association between non-responder status and incident AF in either men or women indicating that exclusion of these individuals most probably did not impact the results of this study. Additionally, stress was treated as a categorical variable due to the nature of the question exploring non-occupational stress. Results did not change when repeating all analyses treating our stress variable as ordinal, or when using only the continuous variable job strain. Second, the inclusion of only hospital-confirmed AF could mean that asymptomatic and less severe AF remained undiagnosed with subsequent underestimation of the true number of individuals affected by neurogenic AF. Moreover, our results cannot be generalized to occurrences where acute stress acts as a trigger of AF episodes as may be the case in specific patients. Third, the characteristics of the study population may limit generalizability to other countries or age-groups. Fourth, information on family history of AF was not available for this cohort. Finally, other studies have also shown statistically non-significant gene-environment interactions^33^. One reason for this may be insufficient statistical power.

This study also has several strengths; it used a large general population cohort relying on national registries with high validity of AF diagnosis while utilizing a genetic risk score known to predict incident AF. Moreover, the study included a validated component for the definition of psychological stress with a resulting composite stress variable which matched specific baseline characteristics of stressed individuals. Moreover, population size was large enough to allow for gender-stratified analyses, and important factor when considering that men and women may differ in their response to stress^28^.

## Conclusion

This is the first study of its kind to investigate the interaction between stress and a genetic risk score strongly predictive of AF. The findings of this study do not directly support an association between chronic psychological stress and the development of incident hospital diagnosed AF, and do not find any interaction between stress and genetic risk of AF.

## Additional Information

**How to cite this article**: Svensson, T. *et al*. Psychological stress and risk of incident atrial fibrillation in men and women with known atrial fibrillation genetic risk scores. *Sci. Rep.* **7**, 42613; doi: 10.1038/srep42613 (2017).

**Publisher's note:** Springer Nature remains neutral with regard to jurisdictional claims in published maps and institutional affiliations.

## Supplementary Material

Supplementary Tables

## Figures and Tables

**Table 1:** Baseline characteristics according to perceived stress level for men and women respectively.

Variable	Men				Women			
	Psychological stress			P-value^a^	Psychological stress			P-value^a^
	Low	Intermediate	High		Low	Intermediate	High	
**Number of individuals**	3806	3813	1146		4392	6393	2758	
**Age [mean (years ± s.d.)]**	60.3 ± 7.2	59.6 ± 7.1	56.9 ± 6.5	<0.0001	57.9 ± 8.2	57.8 ± 8.2	54.9 ± 7.3	<0.0001
**Education (%)**				<0.0001				<0.0001
Primary school or less	42.4	50.0	43.0		35.4	42.4	32.5	
Higher than primary school	57.4	49.6	56.7		64.5	57.5	67.3	
Missing information	0.2	0.4	0.3		0.1	0.1	0.3	
**Socioeconomic Index (%)**				<0.0001				<0.0001
Manual worker	27.2	39.2	33.5		29.8	40.7	34.4	
Lower and intermediate non-manual worker	37.3	29.6	27.6		49.9	42.3	44.9	
Higher non-manual worker	12.6	10.4	9.5		6.8	5.5	6.3	
Other (self-employed and farmers)	18.6	15.0	17.2		10.0	5.8	6.9	
Unemployed	4.1	5.6	12.0		3.2	5.4	7.4	
Missing information	0.3	0.2	0.2		0.3	0.3	0.2	
**Smoking status (%)**				<0.0001				<0.0001
Never	29.9	27.0	25.6		45.2	43.8	38.5	
Past	44.4	43.7	40.1		29.5	28.3	27.1	
Current	25.7	29.3	34.3		25.4	28.0	34.5	
Missing information	0	0	0.1		0	0	0	
**Alcohol consumption (%)**				<0.0001				<0.0001
None	10.0	12.2	16.5		16.3	20.0	20.5	
1–15.2 g ethanol/day	49.0	47.8	41.4		64.8	63.6	60.6	
≥15.2 g ethanol/day	40.0	38.6	39.1		17.9	14.9	16.0	
Missing information	1.0	1.4	3.1		1.1	1.5	2.9	
**BMI [mean (kg/m^2^ ± s.d.)]**	26.2 ± 3.3	26.4 ± 3.6	26.3 ± 3.6	n.s.	25.2 ± 4.0	25.6 ± 4.4	25.5 ± 4.4	<0.0001
**Prevalent diabetes mellitus (%)**	5.5	6.1	6.6	n.s.	2.9	3.9	3.1	<0.0001
**Prevalent coronary event (%)**	4.1	4.0	4.4	n.s.	0.5	0.6	0.6	n.s.
**Prevalent heart failure (%)**	0.4	0.3	0.3	n.s.	0.1	0.1	0.2	n.s.
**Hypertension (%)**	48.0	48.1	46.6	n.s.	36.7	36.9	32.7	0.0004

N.s. = non-significant, s.d. = standard deviation.

^a^Chi-square test for categorical variables, Welch’s ANOVA for continuous variables.

**Table 2:** Cox proportional hazard models for the main effects of an Atrial Fibrillation Genetic Risk Score (AF-GRS) and stress on incident AF.

	AF-GRS Quartiles				Psychological stress		
	Q1	Q2	Q3	Q4	Low	Intermediate	High
**Incident AF (All)**							
Person Years	82,929	84,132	80,486	81,230	120,428	150,221	58,129
No. (Events)	5592 (409)	5662 (465)	5465 (524)	5589 (650)	8198 (839)	10,206 (915)	3904 (294)
Age and gender adjusted model^a^ HR (95% CI)	Reference	1.12 (0.98–1.28)	1.33*** **(1.17–1.52)**	1.69*** **(1.49–1.91)**	Reference	0.97 (0.88–1.06)	1.16* **(1.01–1.32)**
Multivariable model^b^ HR (95% CI)	Reference	1.13 (0.99–1.29)	1.36*** (1.19–1.55)	1.72*** (1.52–1.95)	Reference	0.93 (0.84–1.02)	1.10 (0.96–1.26)
**Incident AF (Men)**							
Person Years	31,533	32,348	30,382	30,367	53,819	54,224	16,587
No. (Events)	2196 (215)	2253 (274)	2147 (281)	2169 (346)	3806 (526)	3813 (461)	1146 (129)
Age adjusted model^c^ HR (95% CI)	Reference	1.26* **(1.05–1.51)**	1.36*** **(1.14–1.62)**	1.71*** **(1.45–2.03)**	Reference	0.93 (0.82–1.06)	1.11 (0.91–1.34)
Multivariable model^d^ HR (95% CI)	Reference	1.25* **(1.05–1.50)**	1.40*** **(1.17–1.67)**	1.75*** **(1.47–2.07)**	Reference	0.89 (0.79–1.02)	1.05 (0.86–1.28)
**Incident AF (Women)**							
Person Years	51,396	51,784	50,104	50,863	66,608	95,997	41,541
No. (Events)	3396 (194)	3409 (191)	3318 (243)	3420 (304)	4392 (313)	6393 (454)	2758 (165)
Age adjusted model^c^ HR (95% CI)	Reference	0.96 (0.79–1.18)	1.31** (1.09–1.58)	1.66*** **(1.39–1.99)**	Reference	1.01 (0.87–1.16)	1.22* **(1.01–1.47)**
Multivariable model^d^ HR (95% CI)	Reference	0.97 (0.80–1.19)	1.31** (1.09–1.59)	1.68*** **(1.40–2.01)**	Reference	0.96 (0.83–1.11)	1.15 (0.95–1.39)

AF-GRS = Atrial fibrillation Genetic Risk Score, CI = Confidence Interval, HR = Hazard Ratio.

^*^p < 0.05; **p < 0.01, ***p < 0.001.

^a^The age and gender adjusted models are adjusted for age at starting point and gender. ^b^The multivariable models are adjusted for age, gender, education, socioeconomic index, smoking, alcohol consumption, prevalent diabetes mellitus, coronary event, heart failure, Body Mass Index, and hypertension.

^c^The age adjusted models are adjusted for age at starting point.

^d^The multivariable models are adjusted for age, education, socioeconomic index, smoking, alcohol consumption, prevalent diabetes mellitus, coronary event, heart failure, Body Mass Index, and hypertension.

**Table 3:** Cox proportional hazard models for the effect of interaction between psychological stress and quartiles of the Atrial Fibrillation Genetic Risk Score (AF-GRS) on incident AF in men and women.

End point	AF-GRS Quartiles				P for interaction^a^
	Q1	Q2	Q3	Q4	
**Incident AF (All)**					
No. (Events)	5592 (409)	5662 (465)	5465 (524)	5589 (650)	
	HR (95% CI)	HR (95% CI)	HR (95% CI)	HR (95% CI)	
**Age and gender adjusted model**^**b**^					
Low stress	Reference	0.96 (0.78–1.19)	1.33** (1.09–1.62)	1.48*** (1.22–1.79)	
Intermediate stress	0.83 (0.67–1.03)	1.10 (0.90–1.34)	1.12 (0.92–1.37)	1.55*** (1.29–1.87)	
High stress	1.08 (0.80–1.44)	1.11 (0.83–1.48)	1.40* (1.07–1.83)	1.94*** (1.51–2.49)	
**Multivariable model**^**c**^					
Low stress	Reference	0.96 (0.78–1.19)	1.35** (1.10–1.64)	1.50*** (1.24–1.82)	
Intermediate stress	0.79* (0.64–0.97)	1.05 (0.86–1.28)	1.11 (0.91–1.35)	1.52*** (1.26–1.83)	
High stress	1.04 (0.78–1.40)	1.09 (0.82–1.46)	1.30 (0.99–1.72)	1.85*** (1.44–2.38)	0.22
**Incident AF (Men)**					
No. (Events)	2196 (215)	2253 (274)	2147 (281)	2169 (346)	
	HR (95% CI)	HR (95% CI)	HR (95% CI)	HR (95% CI)	
**Age adjusted model**^**d**^					
Low stress	Reference	1.01 (0.78–1.30)	1.19 (0.93–1.53)	1.36* (1.07–1.73)	
Intermediate stress	0.71* (0.53–0.94)	1.11 (0.86–1.43)	1.03 (0.80–1.34)	1.40** (1.10–1.79)	
High stress	0.68 (0.42–1.13)	1.07 (0.71–1.61)	1.36 (0.94–1.98)	1.96*** (1.39–2.76)	
**Multivariable model**^**e**^					
Low stress	Reference	1.01 (0.78–1.30)	1.23 (0.96–1.58)	1.41** (1.11–1.79)	
Intermediate stress	0.68** (0.51–0.90)	1.06 (0.82–1.37)	1.04 (0.80–1.34)	1.38** (1.08–1.77)	
High stress	0.69 (0.42–1.14)	1.06 (0.70–1.59)	1.31 (0.90–1.92)	1.85*** (1.30–2.61)	0.17
**Incident AF (Women)**					
No. (Events)	3396 (194)	3409 (191)	3318 (243)	3420 (304)	
	HR (95% CI)	HR (95% CI)	HR (95% CI)	HR (95% CI)	
**Age adjusted model**^**d**^					
Low stress	Reference	0.88 (0.61–1.27)	1.59** (1.15–2.19)	1.71** (1.24–2.35)	
Intermediate stress	1.01 (0.73–1.40)	1.11 (0.81–1.54)	1.27 (0.93–1.75)	1.79*** (1.33–2.42)	
High stress	1.50* (1.01–2.22)	1.21 (0.79–1.84)	1.52* (1.01–2.28)	2.03*** (1.39–2.96)	
**Multivariable model**^**e**^					
Low stress	Reference	0.88 (0.61–1.26)	1.55** (1.12–2.15)	1.70*** (1.23–2.34)	
Intermediate stress	0.95 (0.68–1.32)	1.04 (0.76–1.44)	1.23 (0.89–1.69)	1.70*** (1.26–2.31)	
High stress	1.39 (0.93–2.06)	1.17 (0.76–1.78)	1.37 (0.91–2.05)	1.92*** (1.32–2.81)	0.44

*p < 0.05; **p < 0.01, ***p < 0.001.

^a^Likelihood ratio test for overall interaction term.

^b^Age and gender adjusted models are adjusted for age at starting point and gender. ^c^Multivariable models are adjusted for age, gender, education, socioeconomic index, smoking, alcohol consumption, prevalent diabetes mellitus, coronary event, heart failure, Body Mass Index, and hypertension.

^d^Age adjusted models are adjusted for age at starting point. ^e^Multivariable models are adjusted for age, education, socioeconomic index, smoking, alcohol consumption, prevalent diabetes mellitus, coronary event, heart failure, Body Mass Index, and hypertension.

AF-GRS = Atrial fibrillation Genetic Risk Score, CI = Confidence Interval, HR = Hazard Ratio.
