# Supplementary material for: Psychological stress and risk of incident atrial fibrillation in men and women with known atrial fibrillation genetic risk scores
Source: Sci Rep. 2017 Feb 14;7:42613. doi: 10.1038/srep42613 (PMC5307347; doi:10.1038/srep42613)
Supplement: Supplementary Tables [file srep42613-s1.pdf]

Psychological stress and risk of incident atrial fibrillation in men and women with known atrial fibrillation genetic risk scores

Supplementary tables

Thomas Svensson, MD[1,2,3*], Mariusz Kitlinski, MD, PhD[1,4], Gunnar Engström, MD, PhD[1], Olle Melander, MD, PhD[1,5]

1. Department of Clinical Sciences, Lund University, Skåne University Hospital, SE 205 02 Malmö, Sweden;
2. Department of Global Health Policy, Graduate School of Medicine, The University of Tokyo, 7-3-1 Hongo, Bunkyo-ku, Tokyo 113-0033 Japan
3. Department of Neuropsychiatry, Keio University School of Medicine, 35 Shinanomachi, Shinjuku-ku, Tokyo 160-8582, Japan
4. Department of Cardiology, Skåne University Hospital, Malmö, Sweden
5. Department of Internal Medicine, Skåne University Hospital, Malmö, Sweden

Supplementary Table S1. Odds ratios and confidence intervals for the cross-sectional analyses between psychological stress and risk allele carrier status of the 12 SNPs included in the atrial fibrillation genetic risk score (AF-GRS)

	Men and women			Men			Women		
	Psychological stress			Psychological stress			Psychological stress		
**SNP (Gene)**	Low	Intermediate	High	Low	Intermediate	High	Low	Intermediate	High
**rs3903239 (PRRX1)**									
No. (Individuals with risk allele)	8198 (5963)	10,206 (7335)	3904 (2790)	3806 (2725)	3813 (2740)	1146 (811)	4392 (3238)	6393 (4595)	2758 (1979)
Univariable model OR (95% CI)	Reference	0.96 (0.90-1.02)	0.94 (0.86-1.02)	Reference	1.01 (0.92-1.12)	0.96 (0.83-1.11)	Reference	**0.91* (0.84-0.99)**	0.91 (0.81-1.01)
**rs2200733 (PITX2)**									
No. (Individuals with risk allele)	8198 (1662)	10,206 (1980)	3904 (782)	3806 (761)	3813 (733)	1146 (255)	4392 (901)	6393 (1247)	2758 (527)
Univariable model OR (95% CI)	Reference	0.95 (0.88-1.02)	0.99 (0.90-1.08)	Reference	0.95 (0.85-1.07)	1.15 (0.98-1.34)	Reference	0.94 (0.85-1.03)	0.92 (0.81-1.03)
**rs3807989 (CAV1)**									
No. (Individuals with risk allele)	8198 (6873)	10,206 (8546)	3904 (3261)	3806 (3165)	3813 (3234)	1146 (934)	4392 (3708)	6393 (5312)	2758 (2327)
Univariable model OR (95% CI)	Reference	0.99 (0.92-1.07)	0.98 (0.88-1.08)	Reference	**1.13* (1.00-1.28)**	0.89 (0.75-1.06)	Reference	0.91 (0.82-1.01)	1.00 (0.87-1.14)
**rs10821415 (C9orf3)**									
No. (Individuals with risk allele)	8198 (5354)	10,206 (6693)	3904 (2542)	3806 (2534)	3813 (2509)	1146 (732)	4392 (2820)	6393 (4184)	2758 (1810)
Univariable model OR (95% CI)	Reference	1.01 (0.95-1.08)	0.99 (0.92-1.07)	Reference	0.97 (0.88-1.06)	0.89 (0.77-1.02)	Reference	1.06 (0.97-1.14)	1.06 (0.96-1.18)
**rs10824026 (SYNP02L)**									
No. (Individuals with risk allele)	8198 (8005)	10,206 (9928)	3904 (3809)	3806 (3715)	3813 (3712)	1146 (1110)	4392 (4290)	6393 (6216)	2758 (2699)
Univariable model OR (95% CI)	Reference	0.86 (0.72-1.04)	0.97 (0.75-1.24)	Reference	0.90 (0.68-1.20)	0.76 (0.51-1.12)	Reference	0.84 (0.65-1.07)	1.09 (0.79-1.50)
**rs1152591 (SYNE2)**									
No. (Individuals with risk allele)	8198 (6250)	10,206 (7654)	3904 (2880)	3806 (2891)	3813 (2871)	1146 (848)	4392 (3359)	6393 (4783)	2758 (2032)
Univariable model OR (95% CI)	Reference	0.94 (0.87-1.00)	**0.88** (0.80-0.96)**	Reference	0.97 (0.87-1.07)	0.90 (0.77-1.05)	Reference	**0.91* (0.84-1.00)**	**0.86** (0.77-0.96)**
**rs7164883 (HCN4)**									
No. (Individuals with risk allele)	8198 (2667)	10,206 (3339)	3904 (1265)	3806 (1221)	3813 (1246)	1146 (362)	4392 (1446)	6393 (2093)	2758 (903)
Univariable model OR (95% CI)	Reference	1.01 (0.95-1.07)	0.99 (0.92-1.08)	Reference	1.03 (0.93-1.13)	0.98 (0.85-1.13)	Reference	0.99 (0.91-1.08)	0.99 (0.90-1.10)
**rs2106261 (ZFHX3)**									
No. (Individuals with risk allele)	8198 (2746)	10,206 (3465)	3904 (1259)	3806 (1305)	3813 (1297)	1146 (376)	4392 (1441)	6393 (2168)	2758 (883)
Univariable model OR (95% CI)	Reference	1.02 (0.96-1.09)	0.95 (0.87-1.03)	Reference	0.99 (0.90-1.09)	0.94 (0.81-1.08)	Reference	1.05 (0.97-1.14)	0.96 (0.87-1.07)

Supplementary Table S1 cont.

**rs10033464 (PITX2)**									
No. (Individuals with risk allele)	8198 (1697)	10,206 (2086)	3904 (801)	3806 (781)	3813 (785)	1146 (238)	4392 (916)	6393 (1301)	2758 (563)
Univariable model OR (95% CI)	Reference	0.98 (0.92-1.06)	0.99 (0.90-1.09)	Reference	1.00 (0.90-1.12)	1.02 (0.86-1.20)	Reference	0.97 (0.88-1.07)	0.97 (0.87-1.10)
**rs17570669 (PITX2)**									
No. (Individuals with risk allele)	8198 (8138)	10,206 (10,147)	3904 (3877)	3806 (3775)	3813 (3785)	1146 (1131)	4392 (4363)	6393 (6392)	2758 (2746)
Univariable model OR (95% CI)	Reference	1.27 (0.88-1.82)	1.06 (0.67-1.67)	Reference	1.11 (0.67-1.85)	0.62 (0.33-1.15)	Reference	1.37 (0.82-2.27)	1.52 (0.78-2.99)
**rs3853445 (PITX2)**									
No. (Individuals with risk allele)	8198 (7632)	10,206 (9522)	3904 (3625)	3806 (3541)	3813 (3542)	1146 (1055)	4392 (4091)	6393 (5980)	2758 (2570)
Univariable model OR (95% CI)	Reference	1.03 (0.92-1.16)	0.96 (0.83-1.12)	Reference	0.98 (0.82-1.17)	0.87 (0.68-1.11)	Reference	1.07 (0.91-1.24)	1.01 (0.83-1.22)
**rs13376333 (KCNN3)**									
No. (Individuals with risk allele)	8198 (4659)	10,206 (5721)	3904 (2206)	3806 (2136)	3813 (2149)	1146 (621)	4392 (2523)	6393 (3572)	2758 (1585)
Univariable model OR (95% CI)	Reference	0.97 (0.91-1.03)	0.99 (0.91-1.07)	Reference	1.01 (0.92-1.11)	0.93 (0.81-1.06)	Reference	0.94 (0.87-1.01)	1.00 (0.91-1.10)

*p<0.05; **p<0.01;
AF-GRS= Atrial fibrillation Genetic Risk Score, CI=Confidence Interval, OR=Odds Ratio, SNP=Single Nucleotide Polymorphism

Supplementary table S2. Cox proportional hazard models for the main effects of an Atrial Fibrillation Genetic Risk Score (AF-GRS) and psychological stress on incident AF in men and women without beta receptor antagonists at the start of the study

	AF-GRS Quartiles				Psychological stress		
	Q1	Q2	Q3	Q4	Low	Intermediate	High
**Incident AF (All)**							
Person Years	75,042	75,712	73,585	74,149	109,060	136,136	53,291
No. (Events)	5020 (308)	5052 (356)	4954 (429)	5060 (549)	7340 (665)	9192 (746)	3554 (231)
Age and gender adjusted model[a] HR (95% CI)	Reference	1.14 (0.98-1.33)	**1.42*** (1.22-1.64)**	**1.85*** (1.61-2.12)**	Reference	0.99 (0.89-1.10)	1.14 (0.98-1.32)
Multivariable model[b] HR (95% CI)	Reference	1.13 (0.97-1.32)	**1.42*** (1.23-1.65)**	**1.85*** (1.61-2.13)**	Reference	0.96 (0.86-1.07)	1.08 (0.92-1.26)
**Incident AF (Men)**							
Person Years	28,066	28,467	27,496	27,349	48,057	48,414	14,908
No. (Events)	1932 (160)	1952 (203)	1914 (226)	1936 (291)	3346 (413)	3371 (367)	1017 (100)
Age adjusted model[c] HR (95% CI)	Reference	**1.27* (1.03-1.56)**	**1.41*** (1.15-1.73)**	**1.90*** (1.56-2.30)**	Reference	0.95 (0.82-1.09)	1.09 (0.88-1.36)
Multivariable model[d] HR (95% CI)	Reference	**1.25* (1.01-1.53)**	**1.44*** (1.17-1.76)**	**1.89*** (1.56-2.30)**	Reference	0.92 (0.80-1.06)	1.04 (0.83-1.31)
**Incident AF (Women)**							
Person Years	46,975	47,244	46,088	46,799	61,003	87,722	38,382
No. (Events)	3088 (148)	3100 (153)	3040 (203)	3124 (258)	3994 (252)	5821 (379)	2537 (131)
Age adjusted model[c] HR (95% CI)	Reference	1.00 (0.80-1.26)	**1.43** (1.16-1.77)**	**1.79*** (1.47-2.20)**	Reference	1.05 (0.89-1.23)	**1.20* (0.97-1.48)**
Multivariable model[d] HR (95% CI)	Reference	1.00 (0.80-1.26)	**1.42(* (1.15-1.75)**	**1.80*** (1.47-2.21)**	Reference	1.01 (0.86-1.19)	1.12 (0.90-1.39)

*p<0.05; **p<0.01, ***p<0.001;
[a]The age and gender adjusted models are adjusted for age at starting point and gender
[b]The multivariable models are adjusted for age, gender, education, socioeconomic index, smoking, alcohol consumption, prevalent diabetes mellitus, coronary event, heart failure, Body Mass Index, and hypertension
[c]The age adjusted models are adjusted for age at starting point
[d]The multivariable models are adjusted for age, education, socioeconomic index, smoking, alcohol consumption, prevalent diabetes mellitus, coronary event, heart failure, Body Mass Index, and hypertension
AF-GRS= Atrial fibrillation Genetic Risk Score, CI=Confidence Interval, HR=Hazard Ratio

Supplementary table S3. Cox proportional hazard models for the effect of interaction between psychological stress and quartiles of the AF-genetic risk score (AF-GRS) on incident atrial fibrillation (AF) in men and women without beta receptor antagonists at the start of the study.

End point	AF-GRS Quartiles Q1	Q2	Q3	Q4	P for interaction[a]
**Incident AF (All)**					
No. (Events)	5020 (308)	5052 (356)	4954 (429)	5060 (549)	
	HR (95% CI)	HR (95% CI)	HR (95% CI)	HR (95% CI)	
Age and gender adjusted model[b]					
Low stress	Reference	1.00 (0.78-1.27)	**1.48*** (1.18-1.85)**	**1.64** (1.31-2.04)**	
Intermediate stress	0.89 (0.70-1.13)	1.18 (0.94-1.48)	1.25 (0.99-1.56)	**1.75*** (1.42-2.17)**	
High stress	1.09 (0.77-1.52)	1.10 (0.78-1.54)	**1.38* (1.00-1.89)**	**2.29*** (1.73-3.02)**	
Multivariable model[c]					
Low stress	Reference	0.99 (0.78-1.26)	**1.50*** (1.19-1.87)**	**1.66*** (1.33-2.07)**	
Intermediate stress	0.87 (0.68-1.10)	1.13 (0.90-1.43)	1.23 (0.98-1.55)	**1.71*** (1.38-2.12)**	
High stress	1.05 (0.75-1.47)	1.08 (0.77-1.52)	1.27 (0.92-1.75)	**2.17*** (1.64-2.88)**	0.18
**Incident AF (Men)**					
No. (Events)	1932 (160)	1952 (203)	1914 (226)	1936 (291)	
	HR (95% CI)	HR (95% CI)	HR (95% CI)	HR (95% CI)	
Age adjusted model[d]					
Low stress	Reference	1.08 (0.81-1.46)	1.30 (0.98-1.73)	**1.52** (1.15-2.00)**	
Intermediate stress	0.75 (0.54-1.05)	1.12 (0.83-1.51)	1.12 (0.83-1.50)	**1.65*** (1.25-2.18)**	
High stress	0.78 (0.46-1.34)	1.08 (0.67-1.74)	1.34 (0.86-2.09)	**2.21*** (1.50-3.26)**	
Multivariable model[e]					
Low stress	Reference	1.07 (0.80-1.44)	**1.34* (1.00-1.78)**	**1.55** (1.18-2.05)**	
Intermediate stress	0.74 (0.53-1.04)	1.07 (0.80-1.45)	1.12 (0.83-1.51)	**1.62*** (1.22-2.14)**	
High stress	0.78 (0.45-1.33)	1.09 (0.67-1.76)	1.28 (0.82-2.01)	**2.09*** (1.41-3.10)**	0.53
**Incident AF (Women)**					
No. (Events)	3088 (148)	3100 (153)	3040 (203)	3124 (258)	
	HR (95% CI)	HR (95% CI)	HR (95% CI)	HR (95% CI)	
Age adjusted model[d]					
Low stress	Reference	0.85 (0.56-1.29)	**1.82** (1.26-2.62)**	**1.87*** (1.30-2.69)**	
Intermediate stress	1.08 (0.75-1.57)	1.30 (0.90-1.86)	**1.45* (1.01-2.07)**	**1.93*** (1.37-2.71)**	
High stress	1.43 (0.90-2.82)	1.17 (0.71-1.91)	1.50 (0.94-2.40)	**2.49*** (1.65-3.76)**	
Multivariable model[e]					
Low stress	Reference	0.84 (0.55-1.29)	**1.77* (1.23-2.56)**	**1.86*** (1.29-2.67)**	
Intermediate stress	1.03 (0.71-1.50)	1.22 (0.85-1.76)	1.40 (0.97-2.00)	**1.84*** (1.30-2.60)**	
High stress	1.33 (0.83-2.12)	1.11 (0.70-1.82)	1.33 (0.83-2.14)	**2.35*** (1.55-3.56)**	0.15

*p<0.05; **p<0.01, ***p<0.001;
[a]Likelihood ratio test for overall interaction term
[b]Age and gender adjusted models are adjusted for age at starting point and gender
[c]Multivariable models are adjusted for age, gender, education, socioeconomic index, smoking, alcohol consumption, prevalent diabetes mellitus, coronary event, heart failure, Body Mass Index, and hypertension
[d]Age adjusted models are adjusted for age at starting point
[e]Multivariable models are adjusted for age, education, socioeconomic index, smoking, alcohol consumption, prevalent diabetes mellitus, coronary event, heart failure, Body Mass Index, and hypertension
AF-GRS= Atrial fibrillation Genetic Risk Score, CI=Confidence Interval, HR=Hazard Ratio

Supplementary table S4. Cox proportional hazard models for the main effects of an Atrial Fibrillation Genetic Risk Score (AF-GRS) and job strain on incident AF

	AF-GRS Quartiles				Job strain	
	Q1	Q2	Q3	Q4	Low	High
**Incident AF (All)**						
Person Years	82,929	84,132	80,486	81,230	158,579	170,199
No. (Events)	5592 (409)	5662 (465)	5465 (524)	5589 (650)	10,720 (1013)	11,588 (1035)
Age and gender adjusted model[a] HR (95% CI)	Reference	1.12 (0.98-1.28)	**1.33*** (1.17-1.52)**	**1.69*** (1.49-1.91)**	Reference	1.02 (0.93-1.11)
Multivariable model[b] HR (95% CI)	Reference	1.13 (0.99-1.29)	**1.36*** (1.20-1.55)**	**1.72*** (1.52-1.95)**	Reference	0.98 (0.90-1.07)
**Incident AF (Men)**						
Person Years	31,533	32,348	30,382	30,367	66,644	57,986
No. (Events)	2196 (215)	2253 (274)	2147 (281)	2169 (346)	4673 (611)	4092 (505)
Age adjusted model[c] HR (95% CI)	Reference	**1.26* (1.06-1.51)**	**1.36*** (1.14-1.62)**	**1.71*** (1.45-2.03)**	Reference	0.99 (0.88-1.11)
Multivariable model[d] HR (95% CI)	Reference	**1.26* (1.05-1.50)**	**1.40*** (1.17-1.67)**	**1.75*** (1.47-2.08)**	Reference	0.97 (0.86-1.09)
**Incident AF (Women)**						
Person Years	51,396	51,784	50,104	50,863	91,934	112,212
No. (Events)	3396 (194)	3409 (191)	3318 (243)	3420 (304)	6047 (402)	7496 (530)
Age adjusted model[c] HR (95% CI)	Reference	0.97 (0.79-1.18)	**1.31** (1.09-1.58)**	**1.66*** (1.39-1.99)**	Reference	1.05 (0.92-1.20)
Multivariable model[d] HR (95% CI)	Reference	0.97 (0.80-1.19)	**1.32** (1.09-1.59)**	**1.68*** (1.40-2.01)**	Reference	0.99 (0.87-1.14)

*p<0.05; **p<0.01, ***p<0.001;
[a]The age and gender adjusted models are adjusted for age at starting point and gender
[b]The multivariable models are adjusted for age, gender, education, socioeconomic index, smoking, alcohol consumption, prevalent diabetes mellitus, coronary event, heart failure, Body Mass Index, and hypertension
[c]The age adjusted models are adjusted for age at starting point
[d]The multivariable models are adjusted for age, education, socioeconomic index, smoking, alcohol consumption, prevalent diabetes mellitus, coronary event, heart failure, Body Mass Index, and hypertension
AF-GRS= Atrial fibrillation Genetic Risk Score, CI=Confidence Interval, HR=Hazard Ratio

Supplementary table S5. Cox proportional hazard models for the effect of interaction between job strain and quartiles of the AF-genetic risk score (AF-GRS) on incident atrial fibrillation (AF).

	AF-GRS Quartiles				
End point	Q1	Q2	Q3	Q4	P for interaction[a]
**Incident AF (All)**					
No. (Events)	5592 (409)	5662 (465)	5465 (524)	5589 (650)	
	HR (95% CI)	HR (95% CI)	HR (95% CI)	HR (95% CI)	
Age and gender adjusted model[b]					
Low strain	Reference	1.02 (0.84-1.23)	**1.33*** (1.11-1.59)**	**1.52*** (1.28-1.82)**	
High strain	0.91 (0.75-1.11)	1.13 (0.94-1.36)	**1.23* (1.02-1.47)**	**1.70*** (1.43-2.03)**	
Multivariable model[c]					
Low strain	Reference	1.01 (0.84-1.22)	**1.34** (1.12-1.61)**	**1.54*** (1.29-1.84)**	
High strain	0.87 (0.71-1.05)	1.09 (0.90-1.31)	1.19 (0.99-1.44)	**1.66*** (1.40-1.98)**	0.15
**Incident AF (Men)**					
No. (Events)	2196 (215)	2253 (274)	2147 (281)	2169 (346)	
	HR (95% CI)	HR (95% CI)	HR (95% CI)	HR (95% CI)	
Age adjusted model[d]					
Low strain	Reference	1.05 (0.83-1.33)	1.22 (0.97-1.54)	**1.42** (1.14-1.78)**	
High strain	**0.73* (0.56-0.97)**	1.17 (0.92-1.49)	1.15 (0.91-1.47)	**1.60*** (1.27-2.01)**	
Multivariable model[e]					
Low strain	Reference	1.04 (0.82-1.32)	1.25 (0.99-1.58)	**1.45** (1.16-1.81)**	
High strain	**0.71* (0.54-0.93)**	1.14 (0.89-1.45)	1.16 (0.91-1.48)	**1.59*** (1.26-2.00)**	0.06
**Incident AF (Women)**					
No. (Events)	3396 (194)	3409 (191)	3318 (243)	3420 (304)	
	HR (95% CI)	HR (95% CI)	HR (95% CI)	HR (95% CI)	
Age adjusted model[d]					
Low strain	Reference	0.96 (0.70-1.31)	**1.50** (1.12-2.00)**	**1.69*** (1.28-2.25)**	
High strain	1.13 (0.85-1.50)	1.10 (0.82-1.47)	**1.33* (1.00-1.77)**	**1.85*** (1.41-2.41)**	
Multivariable model[e]					
Low strain	Reference	0.94 (0.69-1.29)	**1.48** (1.11-1.97)**	**1.69*** (1.27-2.24)**	
High strain	1.04 (0.78-1.39)	1.04 (0.78-1.39)	1.25 (0.94-1.66)	**1.74*** (1.33-2.28)**	0.50

*p<0.05; **p<0.01, ***p<0.001;
[a]Likelihood ratio test for overall interaction term
[b]Age and gender adjusted models are adjusted for age at starting point and gender
[c]Multivariable models are adjusted for age, gender, education, socioeconomic index, smoking, alcohol consumption, prevalent diabetes mellitus, coronary event, heart failure, Body Mass Index, and hypertension
[d]Age adjusted models are adjusted for age at starting point
[e]Multivariable models are adjusted for age, education, socioeconomic index, smoking, alcohol consumption, prevalent diabetes mellitus, coronary event, heart failure, Body Mass Index, and hypertension
AF-GRS= Atrial fibrillation Genetic Risk Score, CI=Confidence Interval, HR=Hazard Ratio

Supplementary table S6. Cox proportional hazard models for the main effects of an Atrial Fibrillation Genetic Risk Score (AF-GRS) and non-occupational stress on incident AF

	AF-GRS Quartiles				Non-occupational stress	
	Q1	Q2	Q3	Q4	Low	High
**Incident AF (All)**						
Person Years	82,929	84,132	80,486	81,230	232,498	96,280
No. (Events)	5592 (409)	5662 (465)	5465 (524)	5589 (650)	15,882 (1580)	6426 (468)
Age and gender adjusted model[a] HR (95% CI)	Reference	1.12 (0.98-1.28)	**1.33*** (1.17-1.52)**	**1.69*** (1.49-1.91)**	Reference	1.09 (0.99-1.22)
Multivariable model[b] HR (95% CI)	Reference	1.13 (0.99-1.29)	**1.36*** (1.20-1.55)**	**1.72*** (1.52-1.95)**	Reference	1.07 (0.96-1.19)
**Incident AF (Men)**						
Person Years	31,533	32,348	30,382	30,367	95,219	29,412
No. (Events)	2196 (215)	2253 (274)	2147 (281)	2169 (346)	6752 (902)	2013 (214)
Age adjusted model[c] HR (95% CI)	Reference	**1.26* (1.06-1.51)**	**1.36*** (1.14-1.62)**	**1.71*** (1.45-2.03)**	Reference	1.04 (0.90-1.22)
Multivariable model[d] HR (95% CI)	Reference	**1.26* (1.05-1.50)**	**1.40*** (1.17-1.67)**	**1.75*** (1.48-2.08)**	Reference	0.99 (0.85-1.16)
**Incident AF (Women)**						
Person Years	51,396	51,784	50,104	50,863	137,279	66,867
No. (Events)	3396 (194)	3409 (191)	3318 (243)	3420 (304)	9130 (678)	4413 (254)
Age adjusted model[c] HR (95% CI)	Reference	0.96 (0.79-1.18)	**1.31** (1.09-1.58)**	**1.66*** (1.39-1.99)**	Reference	1.14 (0.99-1.33)
Multivariable model[d] HR (95% CI)	Reference	0.97 (0.80-1.19)	**1.32** (1.09-1.59)**	**1.68*** (1.40-2.01)**	Reference	1.14 (0.98-1.32)

*p<0.05; **p<0.01, ***p<0.001;

[a]The age and gender adjusted models are adjusted for age at starting point and gender

[b]The multivariable models are adjusted for age, gender, education, socioeconomic index, smoking, alcohol consumption, prevalent diabetes mellitus, coronary event, heart failure, Body Mass Index, and hypertension

[c]The age adjusted models are adjusted for age at starting point

[d]The multivariable models are adjusted for age, education, socioeconomic index, smoking, alcohol consumption, prevalent diabetes mellitus, coronary event, heart failure, Body Mass Index, and hypertension

AF-GRS= Atrial fibrillation Genetic Risk Score, CI=Confidence Interval, HR=Hazard Ratio

Supplementary table S7. Cox proportional hazard models for the effect of interaction between non-occupational stress and quartiles of the AF-genetic risk score (AF-GRS) on incident atrial fibrillation (AF)

	AF-GRS Quartiles				
End point	Q1	Q2	Q3	Q4	P for interaction[a]
**Incident AF (All)**					
No. (Events)	5592 (409)	5662 (465)	5465 (524)	5589 (650)	
	HR (95% CI)	HR (95% CI)	HR (95% CI)	HR (95% CI)	
Age and gender adjusted model[b]					
Low strain	Reference	1.12 (0.96-1.30)	**1.34*** (1.16-1.55)**	**1.66*** (1.44-1.91)**	
High strain	1.07 (0.85-1.35)	1.22 (0.98-1.52)	**1.41** (1.14-1.74)**	**1.92*** (1.58-2.33)**	
Multivariable model[c]					
Low strain	Reference	1.13 (0.97-1.31)	**1.39*** (1.20-1.61)**	**1.71*** (1.49-1.97)**	
High strain	1.08 (0.86-1.37)	1.21 (0.97-1.51)	**1.39** (1.12-1.72)**	**1.90*** (1.56-2.31)**	0.91
**Incident AF (Men)**					
No. (Events)	2196 (215)	2253 (274)	2147 (281)	2169 (346)	
	HR (95% CI)	HR (95% CI)	HR (95% CI)	HR (95% CI)	
Age adjusted model[d]					
Low strain	Reference	**1.22* (1.00-1.48)**	**1.28* (1.05-1.56)**	**1.59*** (1.32-1.92)**	
High strain	0.80 (0.55-1.16)	1.20 (0.88-1.64)	**1.41* (1.05-1.91)**	**1.93*** (1.47-2.54)**	
Multivariable model[e]					
Low strain	Reference	**1.22* (1.01-1.49)**	**1.34** (1.10-1.63)**	**1.66*** (1.37-2.00)**	
High strain	0.82 (0.56-1.19)	1.17 (0.86-1.60)	**1.38* (1.02-1.87)**	**1.83*** (1.39-2.42)**	0.60
**Incident AF (Women)**					
No. (Events)	3396 (194)	3409 (191)	3318 (243)	3420 (304)	
	HR (95% CI)	HR (95% CI)	HR (95% CI)	HR (95% CI)	
Age adjusted model[d]					
Low strain	Reference	0.98 (0.77-1.24)	**1.42** (1.14-1.77)**	**1.76*** (1.42-2.18)**	
High strain	1.32 (0.98-1.80)	1.24 (0.90-1.69)	**1.41* (1.04-1.92)**	**1.90*** (1.44-2.52)**	
Multivariable model[e]					
Low strain	Reference	0.99 (0.78-1.25)	**1.43** (1.15-1.79)**	**1.77*** (1.43-2.19)**	
High strain	1.32 (0.97-1.79)	1.24 (0.91-1.69)	**1.39* (1.02-1.89)**	**1.94*** (1.46-2.57)**	0.47

*p<0.05; **p<0.01, ***p<0.001;
[a]Likelihood ratio test for overall interaction term
[b]Age and gender adjusted models are adjusted for age at starting point and gender
[c]Multivariable models are adjusted for age, gender, education, socioeconomic index, smoking, alcohol consumption, prevalent diabetes mellitus, coronary event, heart failure, Body Mass Index, and hypertension
[d]Age adjusted models are adjusted for age at starting point
[e]Multivariable models are adjusted for age, education, socioeconomic index, smoking, alcohol consumption, prevalent diabetes mellitus, coronary event, heart failure, Body Mass Index, and hypertension
AF-GRS= Atrial fibrillation Genetic Risk Score, CI=Confidence Interval, HR=Hazard Ratio
